# Dialysis Disequilibrium Syndrome: A Red Flag to Check Post Hemodialysis

**DOI:** 10.7759/cureus.24619

**Published:** 2022-04-30

**Authors:** Amulya Bellamkonda, Tutul Chowdhury, Malavika Shankar, Nicole Gousy

**Affiliations:** 1 Internal Medicine, One Brooklyn Health System, Brooklyn, USA; 2 Medicine, American University of Antigua, New York, USA

**Keywords:** hemodialysis, rare cause of altered mental status, neurologic manifestation, timing of dialysis initiation, dialysis disequilibrium syndrome

## Abstract

Dialysis disequilibrium syndrome (DDS) is a neurological disorder with varying severity which is primarily caused by the rapid removal of urea during hemodialysis, which was first described in the literature in 1962. Common risk factors are extreme age, high blood urea nitrogen, sudden change in dialysis regimen, presence of other conditions causing cerebral edema, preexisting neurological diseases, and increased permeability of the blood-brain barrier. Understanding these risk factors and preventing the syndrome is crucial as no specific treatment guideline has been established yet. In this case report, we are presenting a case with a conglomeration of clinical attributes suggesting DDS.

## Introduction

Dialysis disequilibrium syndrome (DDS) is attributed to an array of clinical constellations of neurologic manifestation that is observed in a dialysis patient. It could occur during or soon after hemodialysis (HD) due to brain edema developing primarily from the osmotic gradient between the brain and plasma [1]. Rapid HD could result in edematous changes in the brain causing a variety of non-focal neurologic symptoms like alteration of mental status, tremors, convulsions, and in fatal cases even death [2]. Mostly, the reverse urea effect and paradoxical metabolic acidosis are considered as key mechanisms for this extremely rare condition. Numerous guidelines have been established for preventing the development of DDS, making this syndrome increasingly rare. Herein, we report a case where a change in mentation from baseline has been seen after a few sessions of HD in a patient in whom new dialysis was initiated.

## Case presentation

**Figure 1:**
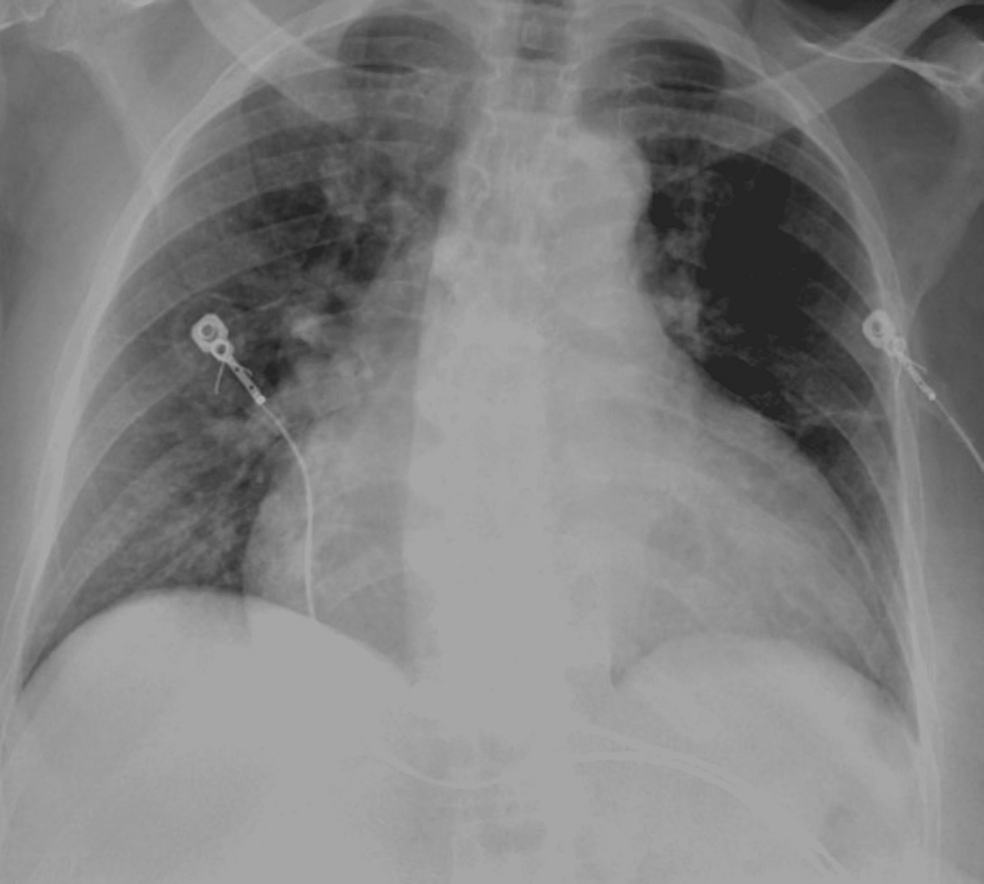
Chest X-ray exhibiting cardiomegaly with mild congestive failure pattern.

A 78-year-old African American female with a past medical history of hypertension and stage 5 chronic kidney disease was referred to ED from the surgery clinic for hypertensive urgency before surgery for dialysis access. Her home medications included carvedilol 6.25 mg twice daily and hydralazine 50 mg thrice daily, however, she admitted to medication noncompliance. On admission, she was awake, alert, and oriented to person, place, and time. Vitals on presentation were 97.7°F, blood pressure was 238/88 mmHg, heart rate was 63 bpm, respiratory rate was 18 breaths/min, with an oxygen saturation of 98% on room air. On receiving hydralazine 20 mg IV push, her systolic blood pressure remained between 190 and 170 mmHg and her diastolic blood pressure remained between 80 and 70 mmHg. Labs on admission were hemoglobin of 9.1 g/dL, hematocrit of 28.6%, blood urea nitrogen (BUN)/CR: 79.9/7.37 mg/dL, potassium of 5.5 mmol/L, sodium of 135 mmol/L, bicarbonate 17 mmol/L, and blood glucose of 97 mg/dL (Table 1). A chest X-ray was taken at this time and showed signs of mild congestive heart failure (Figure 1).

**Table 1:** Trends in electrolytes seen during the course of this patient’s admission. HCT: hematocrit; HGB: hemoglobin; g/dL: grams per deciliter; mcL: microliter; BUN: blood urea nitrogen; GFR: glomerular filtration rate; mL/min: milliliter per minute

	Reference range and units	Admission	Post HD 1st session	Post HD 2nd session	Post HD 3rd session
HBG	11.0-15.0 g/dL	9.1	6.8	7.7	7.4
HCT	35-46 %	28.6	20.2	22.3	22.1
Glucose	80-115 mg/dL	97	90	99	138
BUN	9.8-20.1 mg/dL	79.9	32.6	38.3	17.9
Creatinine	0.57-1.11 mg/dL	7.37	4.92	6.58	4.71
Sodium	136-145 mmol/L	139	135	128	133
Potassium	3.5-5.1 mmol/L	5.5	4.1	4.0	3.5
Chloride	98-107 mmol/L	113	103	92	95
Bicarbonate	23-31 mmol/L	17	25	27	30
Phosphorus	2.5-4.5 mg/dL	6.0	4.3	6.3	4.6
eGFR	>60 mL/min	5.2	8.5	6.0	9.0

**Figure 2:**
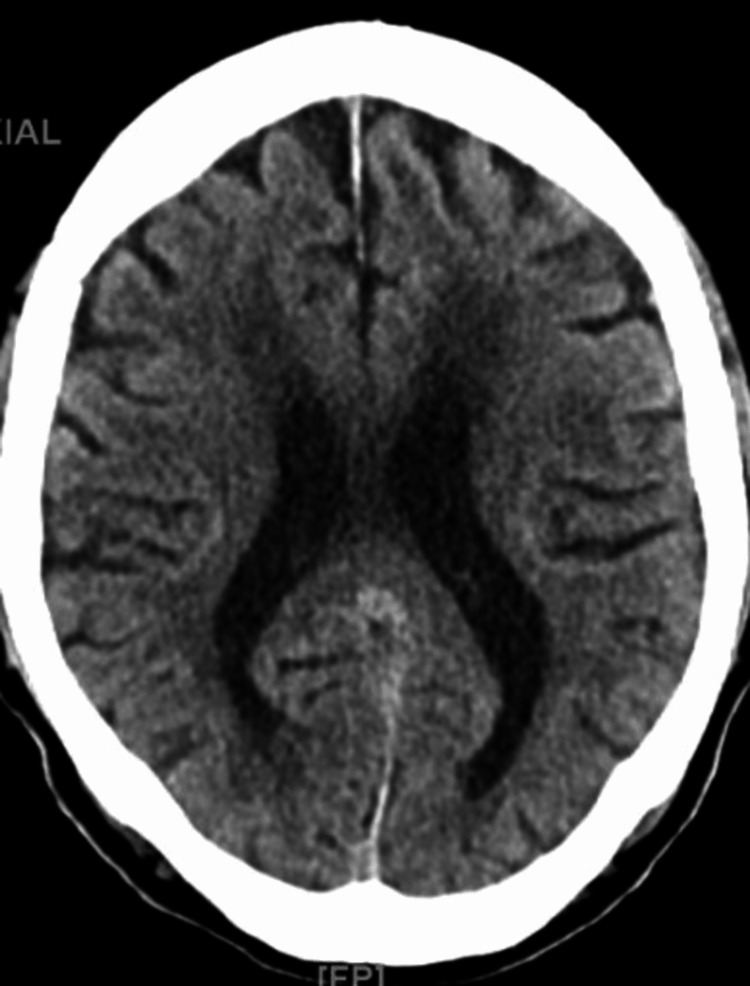
CT head showing bilateral white matter hypodensities compatible with chronic ischemic/degenerative changes, cerebral and cerebellar volume loss, and no acute stroke or evidence of hemorrhage, chronic lacunar infarct in right periventricular white matter is noted measuring up to 6 mm.

On stabilizing blood pressure, an HD access site was created, a right chest permacath and the patient was initiated on her first session of HD with a blood flow rate (BFR) as tolerated to a maximum of 450 mL/min and dialysate flow rate (DFR) of 600 mL/min with no ultrafiltration removed. During admission her medications were adjusted to carvedilol 6.25 mg twice daily, hydralazine 25 mg thrice daily, and minoxidil 5 mg orally to be given post HD on dialysis days. Later on, her blood pressure medications were adjusted according to her blood pressure readings to eventually receiving minoxidil 2.5 mg orally daily and carvedilol 6.25 mg orally twice daily, in addition to discontinuing hydralazine. After the first session of dialysis, the patient was noted to be alert and responsive with occasional periods of disorientation. Meanwhile, her hemoglobin and hematocrit dropped to 6.8 and 20.2, respectively, and the patient received one unit of blood transfusion. She then received a second session of dialysis for 3 hours with net fluid removal of 1 L. The patient tolerated it well and she was awake, alert, and oriented to person, place, and time following the end of her second HD session. Due to the previous drop in hemoglobin levels, a stool occult blood test was done which was negative. The patient had an esophagogastroduodenoscopy (EGD) and colonoscopy to look for internal sources of bleeding followed by the third session of dialysis on the same day for 3 hours and 30 minutes with net fluid removal of 1.5 L. Post the third session of dialysis, a change from baseline mentation was observed. The patient was arousable, communicating coherently, and able to follow simple commands but easily fell back to sleep during the interview with a positive asterixis noted. A CT scan of the head without contrast was repeated and showed no evidence of acute transcortical infarction, acute intracranial hemorrhage, or mass effect (Figure 2). Meanwhile, the patient continued receiving dialysis sessions without ultra-filtrate removal. EGD and colonoscopy results revealed four ulcers 3-7 mm in size in the gastric antrum, and 6 mm sessile polyps in the descending colon (these were later revealed to be hyperplastic polyps on pathology), and diverticulosis. Gastric biopsy results showed positive Giemsa stains for *Helicobacter pylori* organisms. The patient was started on amoxicillin, clarithromycin, and pantoprazole for 2 weeks. A gastrin level was sent and revealed to be 81 pg/mL, which was ruled to be unlikely pathologic. A brain MRI without contrast was also done which identified findings consistent with chronic hypertensive microangiopathy, with no evidence of acute intracranial hemorrhage or edema. The patient was noticed to have significant snoring and, considering her BMI of over 30, obstructive sleep apnea was suspected and arterial blood gas was done (Table 2). Polysomnography was to be considered an outpatient. A trial of continuous positive airway pressure (CPAP) was not done considering her mentation due to the risk of aspiration.

**Table 2:** The results of the arterial blood gas (ABG) during her admission. PCO2: partial pressure of carbon dioxide; FIO2: fraction of inspired oxygen; PO2: partial pressure of oxygen; HCO3: bicarbonate

Component	Reference range and units	Value
pH, arterial	7.35-7.45	7.45
PCO_2_, arterial	35.0-45.0 mmHg	37.6
PO_2_, arterial	80.0-100.0 mmHg	80.2
HCO_3_, arterial	22.0-28.0 mmol/L	25.9
O_2_ saturation, arterial	92.0-98.5%	96.7
FIO_2_	21% room air	21% room air

A bilateral carotid duplex scan of extracranial carotid vessels was done which showed normal carotid arteries. An echocardiogram showed left ventricular hypertrophy with normal systolic function (ejection fraction of 75-80%). Additionally, a left ventricular outflow tract (LVOT) gradient was 25-30 mmHg, and a grade 2 diastolic dysfunction was recognized. Other findings included a pulmonary arterial systolic pressure (PASP) of 45-50 mmHg, along with a dilated left atrium, mild mitral, aortic, and tricuspid regurgitation with minimal pericardial effusion. After the patient’s third session of dialysis, she received dialysis daily thereafter and an improvement in her mentation was noted. She was awake and oriented, however, she remained extremely drowsy and fell asleep with periods of silence followed by loud snoring during assessment intermittently, with a resolution of her asterixis. She was able to withdraw extremities well to a noxious stimulus.

## Discussion

DDS is a rare syndrome defined as a plethora of neurological symptoms seen during or following HD treatment [1-3]. While it most commonly occurs after the first dialysis treatment in newly initiated patients, it can also occur in chronic dialysis patients, those who regularly miss dialysis treatments, or those with acute kidney injury on continuous renal replacement therapy [1,4]. This rare syndrome has a low rate of incidence partly because the corresponding neurological symptoms are so nonspecific that they oftentimes go unnoticed [1,4,5], and partly because recent changes in dialysis prescription protocol have also reduced the incidence of DDS making a diagnosis of DDS in current practice quite unusual. Symptoms of DDS have quite a large spectrum, with milder symptoms such as headache, nausea, and muscle cramps; to more life-threatening symptoms such as seizures, altered mental status, asterixis, and coma [1,3,4]. These symptoms are thought to arise from fluid shifts during HD leading to cerebral edema, hence the wide variety of self-limiting, non-focal neurological symptoms.

This uncommon syndrome may be a clinical diagnosis of exclusion because there are no specific tests for DDS, however, CT scans of the brain may be helpful in exclusion of other potential causes [3]. Risk factors for the development of DDS include the first HD treatment; elevated BUN over 100 mh/dL prior to dialysis initiation; metabolic acidosis; hyperosmolarity due to hyperglycemia or hypernatremia; very old or very young patient ages; preexisting neurological diseases such as stroke, seizure disorder, malignant hypertension; or preexisting conditions leading to increased permeability of the blood-brain barrier such as encephalitis, meningitis, hemolytic uremic syndrome, or vasculitis [1]. While this patient was experiencing non-focal neurologic changes, it was increasingly difficult to find an organic cause precluding these changes making DDS a considerable differential diagnosis during this patient’s admission.

Owing to the rarity of this syndrome, the pathogenesis has not yet been fully mapped out. However, because the symptoms of DDS relate to an osmotic fluid shift into cerebral parenchyma, the rate of dialysis treatment can instigate the development of cerebral edema through several proposed mechanisms: the reserve osmotic shift theory, the intracerebral acidosis theory, and the idiogenic osmol theory [3,4].

The reserve osmolarity shift theory respects the movement of urea from the blood-brain barrier and water during dialysis. In chronically uremic patients, urea is uniformly distributed in brain parenchyma as well as other tissues, making urea an ineffective osmole [4]. However, during dialysis, urea is rapidly removed from peripheral tissues and blood faster than urea can equilibrate out of brain parenchyma. This creates an osmotic gradient that pulls extracellular water into the brain leading to cerebral edema, elevated intracranial pressure, and the neurologic symptoms associated with DDS [3,4]. The study by Kennedy et al. supports this theory as they reported a significant urea gradient between the peripheral blood and cerebrospinal fluid (CSF) in post-dialysis patients [2,4]. Further animal studies showed fluctuations of urea transporters in the brain (UT-B1) and aquaporin channels (AQPs) furthering the validity of this theory [6]. These studies showed that with the rapid removal of urea during HD, there was a decrease in UT-B1 channel expression and an increase in AQP channels on the blood-brain barrier [4,6]. The changes in these channels propagate urea trapping in the brain, therefore, potentiating the osmotic gradient, while also facilitating the development of interstitial cerebral edema seen in DDS via improved water flow into the brain [3,4,6].

The intracerebral acidosis theory considers the use of dialysate during HD to potentially reduce preexisting metabolic acidosis in a patient undergoing HD. Since dialysate contains bicarbonate, the use of this solute can raise the pH of the blood enough to potentially induce compensatory hyperventilation. When hyperventilation occurs, bicarbonate is converted to carbon dioxide via carbonic anhydrase [3,4]. The carbon dioxide is then freely diffused into the brain and CSF forming carbonic acid, therefore decreasing CSF pH. This leads to the displacement of sodium and potassium ions from bound proteins, therefore making them osmotically active, propagating cerebral edema [3,4]. Lastly, the idiogenic osmol theory suggests that after a rapid correction of hypernatremia or hyperglycemia, idiogenic osmoles are created as an adaptive reflex from the cerebral cortex. These osmoles create the osmotic gradient that leads to cerebral edema and the development of DDS [6,7].

Since this diagnosis is one of exclusion, another similarly presenting disease must be taken into consideration: posterior reversible encephalopathy syndrome (PRES) [8]. PRES is another rare neurologic syndrome that encompasses a variety of neurologic symptoms, including but not limited to seizures, headaches, altered mental status, changes in vision or hearing, focal neurological deficits, with seizures being the most common symptom of PRES [8]. While this can be similar to DDS, it is important to note that while seizures may occur, they are not as common as they are in PRES. Additionally, the neurologic deficits of DDS generally are non-focal while the neurological deficits in PRES are more focal due to specific areas of swelling seen in brain MRI [8]. PRES can be triggered acutely by a variety of factors such as extreme blood pressure fluctuations, eclampsia, autoimmune diseases, renal failure, or exposure to cytotoxic agents including chemotherapeutic agents or immunosuppressive medications [8].

While this patient initially presented with significantly elevated blood pressures, her neurologic symptoms only appeared after the start of her dialysis, which was several days after the resolution of her hypertensive emergency. This made the conclusion of DDS more likely than PRES in this patient. The cornerstone finding on imaging for PRES is vasogenic edema on CT or MRI. Typically, MRI will reveal bilateral vasogenic edema specifically in the parieto-occipital regions [8]. In this patient, however, no areas of significant swelling were identified on head CT or MRI, again pointing to a diagnosis of DDS which can have either nonspecific CT or MRI changes, or generalized cerebral edema [3,4]. Additionally, PRES will resolve acutely after the offending agent or etiology has been rectified, which is not the case for DDS where the symptoms tend to resolve gradually over time as in this patient.

Due to the rarity of DDS, there has not been any treatment protocol to date, however, supportive steps to manage the symptoms of DDS are suggested. Nevertheless, there has been a significant focus on the prevention of DDS by altering the BFR and duration in those that are more susceptible to the development of DDS. Prevention of DDS can be accomplished using three major strategies: 1) reducing the clearance rate to ensure no significant osmotic gradient between CSF and peripheral blood occurs; 2) prolonging the length of dialysis treatments and slowing the BFR to 150-250 mL/min to promote better equilibrium; 3) adding another osmotically active agent such as sodium or mannitol to replace plasma urea so that there is no significant change in plasma osmolarity [1,7]. If patients do not develop DDS after several HD treatments while following these guidelines, BFR and length of dialysis treatment can gradually be increased [1,3,4]. Since these strategies have been a rule of thumb in initiating dialysis in those with newly diagnosed end-stage renal disease, the incidence of DDS has since decreased. In our patient, many other organic causes of her sudden change in altered mental status were investigated with several different modalities of testing. Due to each test proving to be either negative or unlikely to be the source of her sudden non-focal neurologic symptoms, a diagnosis of DDS was considered.

## Conclusions

Although DDS is a rare and self-limiting syndrome, it’s important to be aware of this diagnosis, particularly in high-risk groups. Prevention and early detection of this entity will limit potential serious consequences such as coma and death. Given its complex presentation and lack of awareness among physicians, DDS should be considered in dialysis patients whether it’s newly initiated or in patients who frequently miss dialysis sessions. There have been very few cases reported in whom no structural disorders in the brain are seen, but still developed DDS during the first few sessions of HD even with a gentle dialysis prescription.
